# A Low-Cost CMOS Programmable Temperature Switch

**DOI:** 10.3390/s8053150

**Published:** 2008-05-15

**Authors:** Yunlong Li, Nanjian Wu

**Affiliations:** National Laboratory for Superlattices and Microstructures, Institute of Semiconductors, Chinese Academy of Sciences, P.O.Box 912, Beijing 100083, P. R. China

**Keywords:** temperature switch, floating gate neural MOS, threshold temperature, process compensation

## Abstract

A novel uncalibrated CMOS programmable temperature switch with high temperature accuracy is presented. Its threshold temperature *T*_th_ can be programmed by adjusting the ratios of width and length of the transistors. The operating principles of the temperature switch circuit is theoretically explained. A floating gate neural MOS circuit is designed to compensate automatically the threshold temperature *T*_th_ variation that results form the process tolerance. The switch circuit is implemented in a standard 0.35 μm CMOS process. The temperature switch can be programmed to perform the switch operation at 16 different threshold temperature *T*_th_s from 45—120°C with a 5°C increment. The measurement shows a good consistency in the threshold temperatures. The chip core area is 0.04 mm^2^ and power consumption is 3.1 μA at 3.3V power supply. The advantages of the temperature switch are low power consumption, the programmable threshold temperature and the controllable hysteresis.

## 1. Introduction

Electrical apparatus tend to become smaller in size and more powerful in function with the progress of the microelectronics and semiconductor fabrication technology. However, the power consumption density in an integrated circuit (IC) chip and the electrical apparatus increases rapidly so that the thermal effects in them become more serious. In order to make the IC chip and apparatus operate safely and stably, now a temperature switch is widely used to monitor the temperature of the apparatus and to control their power consumption (thermal-protection). How to realize a programmable temperature switch with high performance, small size and low power consumption is an important study issue. This paper presents a novel uncalibrated CMOS programmable temperature switch with high temperature accuracy.

Thermistor and memory alloy switches are traditional critical temperature switches based on thermal-sensitive materials. These switches have disadvantages of low precision and high cost. They can not be integrated into IC chip with a standard silicon CMOS process. On the other hand, some CMOS temperature switches have been reported. These temperature switches usually consists of a temperature sensor and a comparator. The temperature sensor outputs a voltage (or current) signal which is proportional to absolute temperature (PTAT); then the comparator compares the signal with a reference voltage (or current) and outputs a logic signal. The value of the reference voltage determines threshold temperature *T*_th_ of the switch [1]. The circuits in the temperature switches are complicated and have large power consumption. Recently a new type of subthreshold CMOS temperature switch was reported [2]. The temperature switch is simpler and its power consumption is lower. But, one of its disadvantages is that the threshold temperature *T*_th_ varies evidently with process variation. Another one of its disadvantages is that *T*_th_ hysteresis is too large when the temperature is first increased and then is decreased.

This paper proposes a novel threshold temperature *T*_th_ programmable uncalibrated(need not to be uncalibrated after fabrication) CMOS temperature switch with high temperature accuracy. It consists of a basic temperature switch, a threshold temperature setting module and a sampling and readout module. The temperature switch has some advantages: 1) the circuit structure is simple; 2) its power consumption is low; 3) the threshold temperature can be controlled and programmed and 4) the hysteresis of *T*_th_ can be controlled. The paper is organized as follows. Section 2 gives the temperature switch circuitry and describes its operating principles. In Section 3, the implementation of the temperature is described. Section 4 shows the measurement method and measurement results. Finally, the conclusions are given.

## 2. The Programmable CMOS Temperature Switch

**Figure 1.**
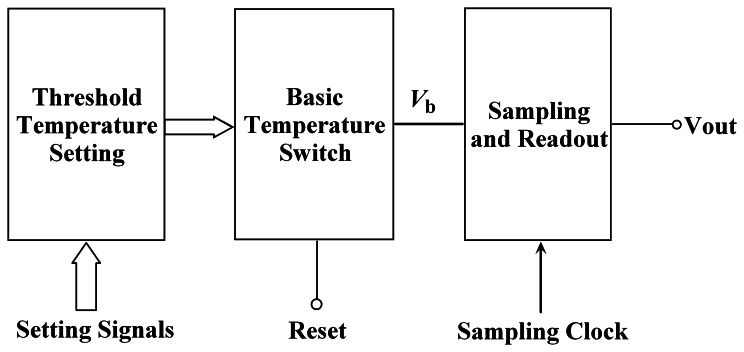
Block diagram of the proposed temperature switch.

Figure 1 shows a block diagram of the proposed CMOS temperature switch. It consists of a basic temperature switch circuit, a threshold temperature setting module and a sampling and readout module. The operation of the basic temperature switch circuit depends on chip temperature, and changes abruptly when the temperature increases to a critical temperature. The critical temperature is called as the threshold temperature *T*_th_ that can be programmed. The basic temperature switch circuit can automatically compensate *T*_th_ variation due to process tolerance. The threshold temperature setting module is used to control the value of *T*_th_ by an external threshold setting signal. The sampling and readout module samples a switch signal and then outputs a digital signal at a fixed frequency.

### 2.1. The Basic Temperature Switch Circuit and Threshold Temperature T_th_

**Figure 2.**
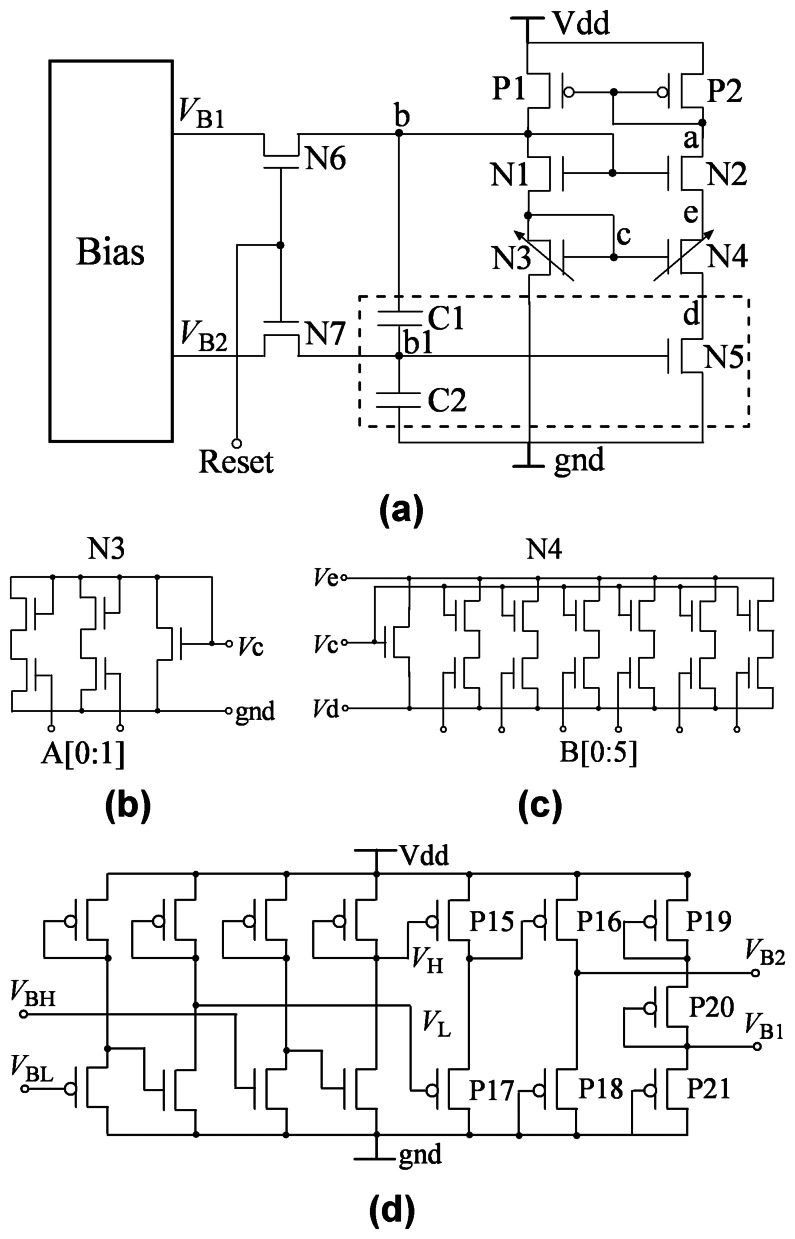
(a) The schematic of the basic temperature switch; (b) the actual schematic of the equivalent N3 transistor; (c) the actual schematic of the equivalent N4 transistor; (d) the schematic of the *V*_B1_ and *V*_B2_ bias circuit that provides biases *V*_B1_ and *V*_B2_ for the floating gate neural MOS circuit.

The basic temperature switch circuit is shown in Figure 2 (a). It consists of two PMOS transistors P1 and P2, seven NMOS transistors N1, N2, N3, N4, N5, N6 and N7, two capacitors C1 and C2, and a bias voltage circuit. The P1, P2, N1, N2, N3, N4 and N5 transistors constitute the temperature switch core circuit. It is similar to the β multiplier circuit [3]. But, the linear resistor in the multiplier circuit is substituted by the transistor N5 [2]. N3 and N4 represent two equivalent nMOS transistors whose ratios of width and length can be adjusted by logic signals from the threshold temperature setting module, respectively. Their actual schematics of the equivalent N3 and N4 transistors are shown in Figure 2 (b) and (c). The transistor N5 and two capacitors C1 and C2 constitute equivalently a floating gate MOS circuit and the transistors N6 and N7 are switches that are controlled by periodical signal RESET. The operation is as follows. First the N6 and N7 transistors are switched on, the voltages Vb and Vb1 at the nodes b and b1 are preset to be equal to VB1 and VB2, respectively. Then N6 and N7 are switched off and the circuit transit into a stable state automatically. When the temperature is lower than *T*_th_, in the final stable state, the transistors P1 and P2 operate in saturation region, while transistors N1, N2, N3 and N4 in subthreshold region, transistor N5 in linear region and *V*_b_ and *V*_b1_ maintain at about several hundred milivoltage. As long as the temperature increases and exceeds to *T*_th_, the final state of the basic temperature switch circuit changes abruptly: the MOS transistors (P1, P2, N1-N5) cut off, and *V*_b_ and *V*_b1_ drop almost to zero. The threshold temperature *T*_th_ of the switch can be preset by adjusting the ratios of width and length of N3 and N4 transistors. The bias voltages *V*_B1_ and *V*_B2_ are supplied by the bias voltage circuit, shown in Fig. 2(d). *V*_B1_ is not dependent on the process tolerance and the chip temperature; *V*_B2_ depends on the threshold voltage variation of the MOS transistor and has a very small temperature coefficient. The bias voltage circuit is used for resetting the temperature switch circuit periodically and compensating for *T*_th_ variation due to process tolerance

The basic relationship among *V*_b_, *V*_b1_ and *T* is presented in[4]. Considering the body-effect and temperature effect, the relationship among *V*_b_, *V*_b1_ and *T* can be rewritten as

(1) $$\exp\left[A_{1}\frac{V_{b}+\left(2+\gamma /2\right)K_{T}T}{T}\right]\times \exp\left[\frac{A_{2}}{T}\right]=A_{3}\ln K_{43}\times \frac{V_{b1}-V_{TH0}+K_{T}T}{T}+A_{4}{\left(\ln K_{43}\right)}^{2}$$

where $A_{1}=\frac{q}{\left(2+\gamma /2\right)\xi K}$, $A_{2}=-\frac{qV_{TH0}}{\xi K}$, $A_{3}=\frac{\xi q\times {K_{31}}^{{\left(2+\gamma /2\right)}^{-1}}}{\left(\xi -1\right)K_{35}K}$, $A_{4}=-\frac{\xi^{2}{K_{31}}^{{\left(2+\gamma /2\right)}^{-1}}}{2\left(\xi -1\right)K_{35}}$, *γ* is the body-effect coefficient, *V*_TH0_ is the zero-bias threshold voltage at absolute zero temperature, *K*_T_ is the zero-bias threshold voltage temperature coefficient (to simplify the analysis, *K*_T_ here equals −*K_T_*_1_/*T_NORM_* in [5]), *T* is the temperature, *K* is Boltzmann constant, *ξ* is the subthreshold swing parameter, *W* and *L* are the channel width and length respectively, *K_ij_* = (*W*/*L*)*_i_* /(*W*/*L*)*_j_* and the footnote numbers represent transistor numbers.

If the floating gate MOS circuit is not used(taking out the capacitors C1 and C2) and *V*_b_ is connected with *V*_b1_ directly(just as [2,6]), differentiating both sides of Eq. (1) with *T*, the derivative of voltage *V*_b_ w.r.t temperature is given by

(2) $$\frac{dV_{b}}{dT}=\frac{\exp\left[\frac{A_{1}V_{b}+A_{1}\left(2+\gamma /2\right)K_{T}T+A_{2}}{T}\right]\;(\frac{A_{1}V_{b}+A_{2}}{T^{2}})-\frac{A_{3}\ln K_{43}.\;(V_{b}-V_{TH0})}{T^{2}}}{\left\{\exp\left[\frac{A_{1}V_{b}+A_{1}\left(2+\gamma /2\right)K_{T}T+A_{2}}{T}\right]\times \frac{A_{1}}{T}-\frac{A_{3}\ln K_{43}}{T}\right\}}$$

The denominator in Eq. (2) decreases with temperature. At the threshold temperature *T*_th_, the denominator becomes to be 0 and the differentiation of the voltage *V*_b_ becomes negative infinity. This indicates that at the threshold temperature *T*_th_ the circuit performs a switching operation. Assuming that the denominator in Eq. (2) equals 0, we can get

(3) $$V_{b}=\frac{T\ln\left(A_{3}\ln K_{43}/A_{1}\right)-A_{2}-A_{1}\left(2+\gamma /2\right)K_{T}T}{A_{1}}$$

Substitute Eq. (3) into Eq. (1), *T*_th_ can be given by

(4) $$T_{th}=\frac{\left(1+\frac{\gamma }{2}\right)V_{TH0}}{\frac{1}{A_{1}}+(1+\frac{\gamma }{2})K_{T}-\frac{A_{4}}{A_{3}}\ln K_{43}-\frac{1}{A_{1}}\ln\frac{A_{3}\ln K_{43}}{A_{1}}}$$

**Figure 3.**
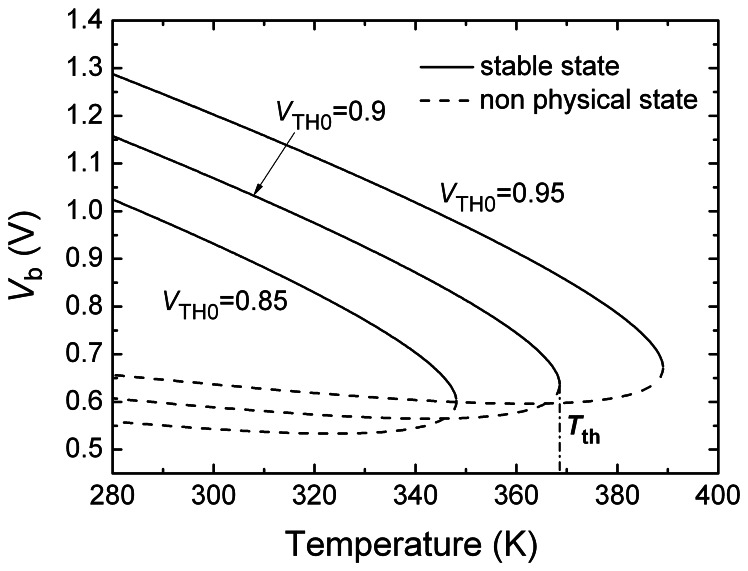
The dependence of *V*_b_ on temperature *T* with three different threshold voltages. It's calculated based on Eq. (6). ξ=1.65, γ=0.7V1/2, KT=1.1mV/K, K43=2, K31=1.4 and K35=560.

In Eq. (4) the derivative of *T*_th_ w.r.t *V*_TH0_ is larger than zero, so *T*_th_ increases linearly with *V*_TH0_ and the slope is very sharp. Figure 3 shows the calculated dependence of *V*_b_ on temperature *T* with three different threshold voltages. It indicates that the threshold temperature *T*_th_ depends strongly on the threshold voltage of MOS transistor. A *V*_TH0_ variation of 50mV results in a *T*_th_ change of 20 K. In these calculations we used the following conditions: *ξ*=1.65, *K*_43_=2, *γ*=0.7 V^1/2^, *K*_T_ =1.1mv/K. The curves show that there are two solutions for a certain temperature only if the temperature is lower than a critical temperature. The solution with a larger *V*_b_ corresponds to the stable operating state of the switch circuit. The transistor N1, N2, N3 and N4 operate in the subthreshold region. The solution with a small smaller *V*_b_ is a pure mathematic one and does not correspond to the physical state of the switch circuit. It is not taken into account in the study. When the temperature increases to and exceeds the critical temperature, the operation of the circuit changes suddenly and all of the transistors N1, N2, N3, N4, N5, P1 and P2 cut off so that no solution can be obtained by the above equations and *V*_b_ becomes almost zero. The critical temperature is considered as the threshold temperature *T*_th_. The N1, N2, N3 and N4 transistors in the switch are all in the subthreshold region, so the currents through them are quite small and power consumption is very low.

### 2.2. Compensation for T_th_ Variation

Equation (4) and Figure 3 show the strong dependence of *T*_th_ on *V*_TH0_. This indicates that the variation of *V*_TH0_ results in a large warp of *V*_b_ (or *V*_b1_), and then makes *T*_th_ change so that the temperature switch does not operate well. If the warp of *V*_b1_ could be compensated, *T*_th_ may keep independent on variation of *V*_TH0_. We design a floating gate neural MOS circuit[7] to compensate *T*_th_ variation. The circuit is surrounded by a dashed line box in Figure 2 (a). If the switches N6 and N7 are on, biases *V*_B1_ and *V*_B2_ preset the voltages at nodes b and b1, respectively. If the ratio of C1 and C2 is *M*, *V*_b1_ equals *V_B_*_2_ −(*V_B_*_1_ − *V_b_*) . *M*/(*M* + 1). Substitute it into Eq. (1) and repeat the derivation as the above, we can get:

(5) $$T_{th}=\frac{\left(M+1\right)V_{B2}-MV_{B1}+\left[M\left(1+\frac{\gamma }{2}\right)-1\right]V_{TH0}}{\frac{M}{A_{1}}-\frac{A_{4}}{A_{3}}\left(M+1\right)\ln K_{43}+K_{T}\left[M\left(1+\frac{\gamma }{2}\right)-1\right]-\frac{M}{A_{1}}\ln\frac{A_{3}M\ln K_{43}}{A_{1}\left(M+1\right)}}$$

(6) $$V_{b}=\frac{T_{th}\ln\left(\frac{A_{3}\ln K_{43}}{A_{1}}•\frac{M}{M+1}\right)-A_{2}-A_{1}(2+\frac{\gamma }{2})K_{T}T_{th}}{A_{1}}$$

**Figure 4.**
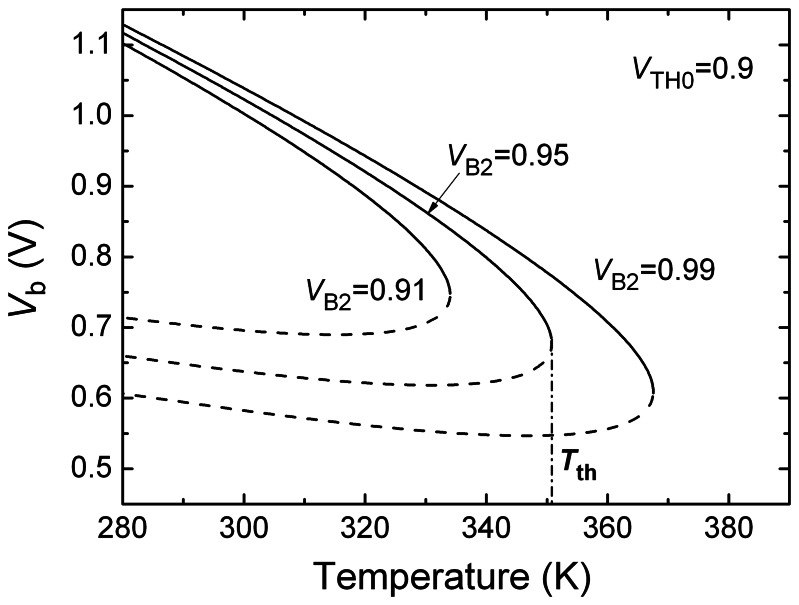
The dependence of *T*_th_ on *V*_B2_ It's calculated based on Eq. (6) and *V_b_*_1_ = *V_B_*_2_ −(*V_B_*_1_ − *V_b_*) . *M*/(*M* + 1) .*ξ*=1.65, *γ*=0.7V^1/2^, *K*_T_ =1.1mV/K, *K*_43_=2, *K*_31_=1.4, *K*_35_=560 and *M*=38/11.

It is found that *V*_b_ and *T*_th_ both increase with *V*_B2_ when other parameters keep invariable. Figure 4 shows the dependence of *T*_th_ on *V*_B2_ with *V*_B1_=1.1V. If we design a bias circuit to make *V*_B1_ remain almost invariable with *V*_TH0_ and *V*_B2_ vary suitably with *V*_TH0_, the *T*_th_ variation could be compensated by presetting the voltages of node b and b1 with *V*_B1_ and *V*_B2_. Figure 2(d) shows the schematic of the *V*_B1_ and *V*_B2_ bias circuit that provides biases *V*_B1_ and *V*_B2_ for the floating gate neural MOS circuit. If we optimize the sizes of transistors reasonably, *V*_H_ and *V*_L_ can vary linearly with *V*_TH0_ and their temperature coefficients are almost same. P15, P16, P17 and P18 constitutes a subtracter [8] and make *V*_B2_ equal *V*_H_ − *V*_L_. *V*_B2_ varies linearly with *V*_TH0_ and its temperature coefficient is small. On the other hand, *V*_B1_ almost does not change with *V*_TH0_ and temperature. It can be obtained by using transistors P19, P20 and P21 to divide the power supply voltage.

**Figure 5.**
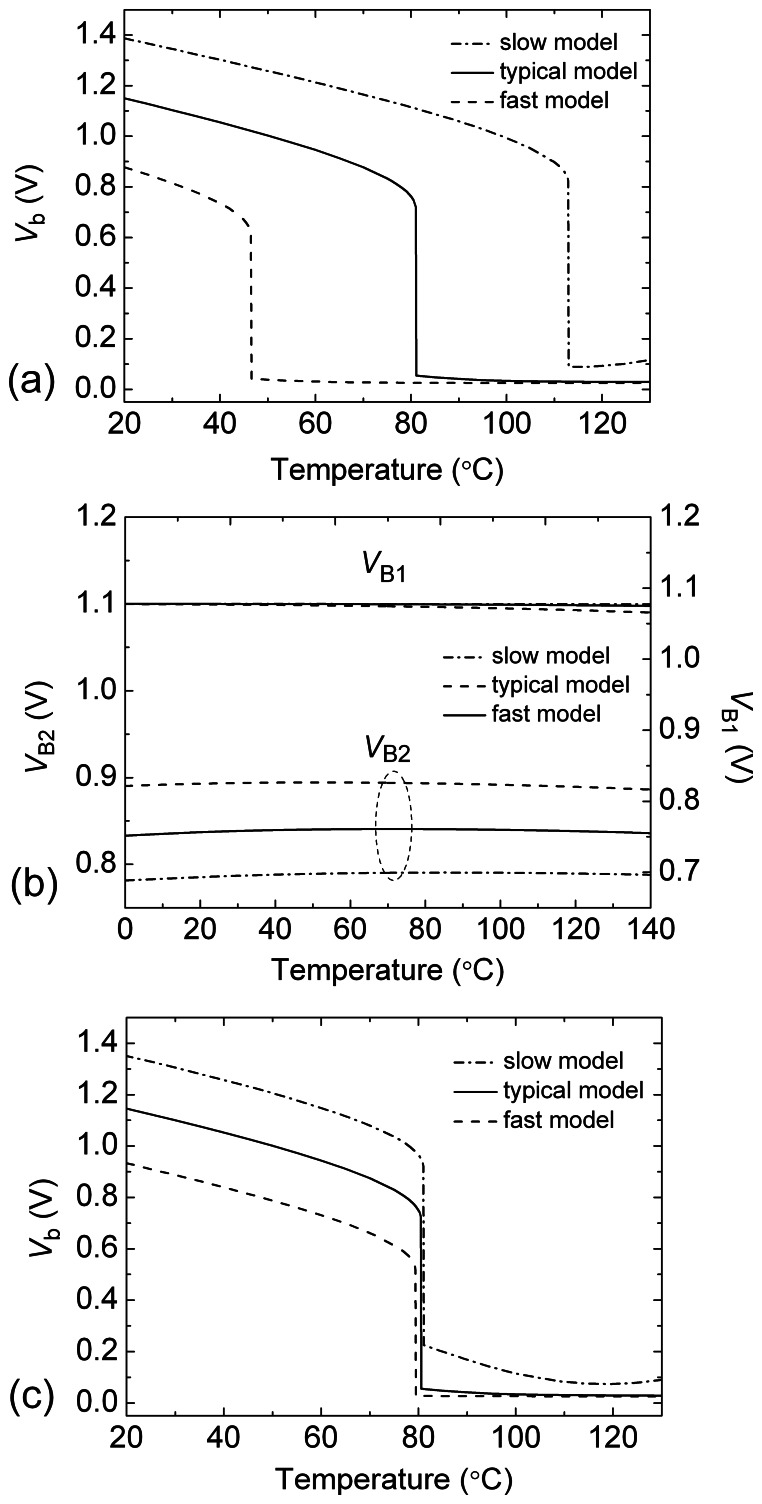
(a) The simulated dependence of *V*_b_ on temperature *T* with three different models before compensation. *V*_B1_=1.1V and *V*_B2_=0.84V. (b) The simulated dependence of *V*_B1_ and *V*_B2_ on temperature *T* with three different models. (c) The simulated dependence of *V*_b_ on temperature *T* with three different models after compensation. *V*_B1_ and *V*_B2_ are supplied by the bias circuit.

We simulated the performance of the temperature switch circuit with three transistor models corresponding to three process corners: slow model, typical model and fast model. It is considered that the effect of *V*_TH0_ variation on *T*_th_ is dominant when the transistor model is changed. Figure 5 (a) shows the *T*_th_ variation that is originated by different process corners without compensation. The *T*_th_ variation is larger than 60°C. Figure 5 (b) shows the dependences of *V*_B1_ and *V*_B2_ on the temperature and the process corner. *V*_B1_ does not change almost with the process corner and the temperature. On the other hand, *V*_B2_ varies linearly with the process corner and its temperature coefficient is small. Figure 5(c) shows the switch characteristics of the temperature switch circuit with the compensation operation in three process corners. We can see that the compensation for the process corner or *V*_TH0_ variation is very effective.

### 2.3. Control of hysteresis

**Figure 6.**
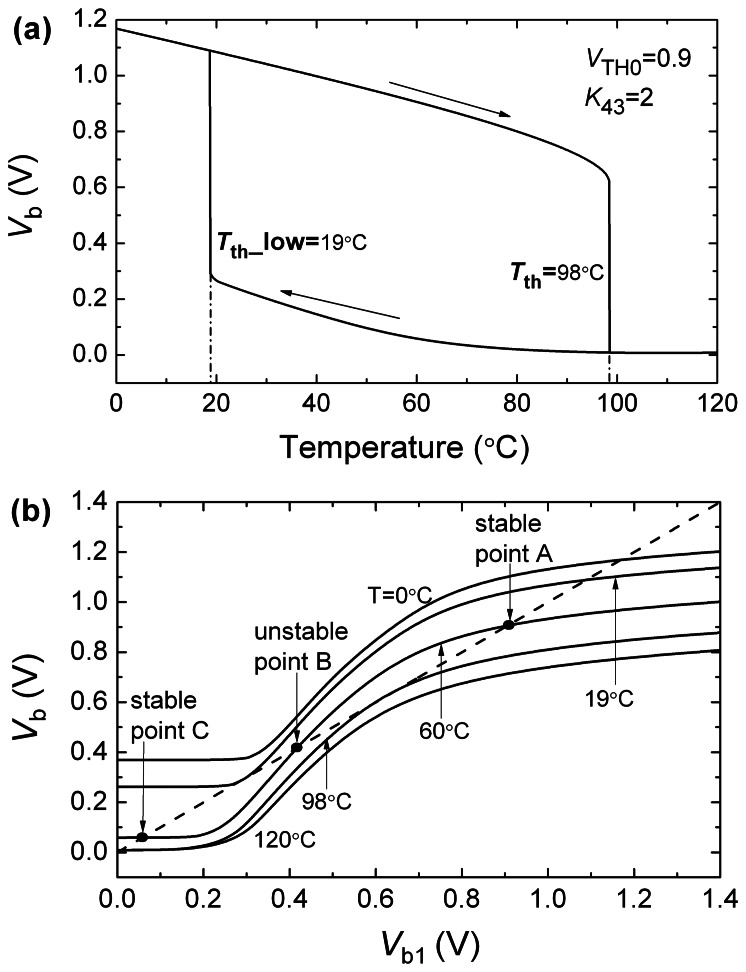
(a) Dependence of *V*_b_ on the chip temperature. The temperature switch shows hysteretic characteristic. It's the simulation result of the temperature switch core circuit with the node b and b1 connecting directly; (b) the dependence of *V*_b_ on *V*_b1_ at different temperatures and *V*_b1_ straight line. The *V*_b_ curve intersects *V*_b1_ line at three points of A, B and C.

We use HSPICE to simulate the operation of the temperature switch circuit. Figure 6(a) gives the dependence of *V*_b_ on the chip temperature. The *T* dependence of *V*_b_ shows the hysteresis behavior reported also by [6]. First *V*_b_ decreases with the increase of the temperature. When the temperature increases to *T*_th_ = 98°C, *V*_b_ abruptly drops down to a lower voltage. Then, when the temperature is decreased from the high temperature and becomes lower than *T*_th_, *V*_b_ does not rebound to a higher voltage until the temperature decreases to a temperature point of 19°C which is much lower than *T*_th_.

This hysteresis phenomenon can be analyzed as follows. Assuming that the nodes b and b1 are opened and that a voltage *V*_b1_ is biased at node b1, the voltage *V*_b_ will change with *V*_b1_. Figure 6(b) shows the dependence of *V*_b_ on *V*_b1_ at different temperatures and *V*_b1_ straight line. If the b and b1 nodes are connected and *V*_b_ is equal to *V*_b1_ in the temperature switch circuit, the intersections of *V*_b_ curves and *V*_b1_ straight line can be considered as the operating points of the switch circuit. At the temperature of 60°C, the *V*_b_ curve intersects *V*_b1_ line at three points of A, B and C. The A and C points correspond to the stable operating points, and B is an unstable point. If the initial *V*_b_ is higher than the voltage of the point B, the circuit will settle down to the stable point A. In contrast, if the initial *V*_b_ is lower than the voltage of the point B, it will settle down to point C. Therefore, if the initial *V*_b_ is higher than the voltage of the point B and the temperature is increased gradually, the switch circuit changes its operating state along a series of A points. When the temperature increase to and exceeds *T*_th_ of 98°C, the *V*_b_ curve intersects *V*_b1_ line only at the C point so that the switch circuit shifts suddenly its state from A to C point and shows a switching operation. If the temperature decreases from a temperature higher than *T*_th_, the circuit operates at the point C because *V*_b_ is smaller than the point B. When the temperature decreases to and is under a critical point of 19°C, the C point disappears and the switch circuit shifts suddenly its operating state from C to A point. Thus, the switch circuit shows the hysteresis behavior which is too large to perform the temperature switch operation well.

How to control the hysteresis is a study issue. We propose a simple method to remove the hysteresis equivalently or to change hysteresis. A bias and resetting circuit is designed in the basic temperature switch circuit, as shown in Figure 2(a). The bias and resetting circuit consists of a V_B1_ and V_B2_ bias voltage circuit, the N6 and N7 resetting-switch transistors and a resetting signal RESET. If the RESET signal is periodic and synchronizes with the operation of sampling and readout module, *V*_b_ will be preset periodically to make the circuit operate at the A operating point initially and the switch operation of the circuit can be measured correctly without a hysteresis. Furthermore, a suitable hysteresis is often required in a practical application. We can integrate two switch circuits with different *T*_th1_ and *T*_th2_ (*T*_th1_ < *T*_th2_) and realize a temperature switch with a hysteresis of Δ*T* = *T*_th2_ - *T*_th1_. The sampling and readout module measures the *V*_b_ voltages of the two switch circuits and outputs a switch signal and a rebound signal when *T* ≥*T*_th2_ and *T* < *T*_th1_, respectively.

### 2.4. Programmable T_th_

**Figure 7.**
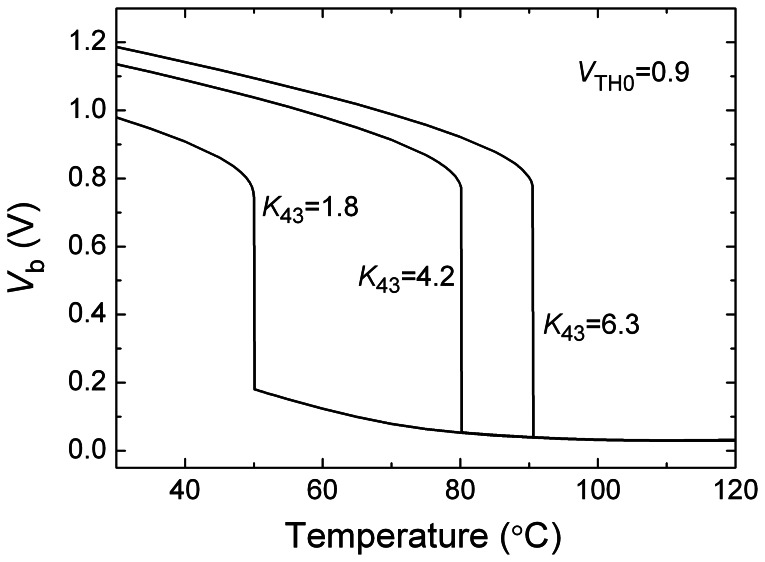
Voltage *V_b_* as a function of temperature with different *K*_43_ parameters.

In practice it is always expected that the threshold temperature *T*_th_ of the proposed temperature switch can be programmed. Equation (4) indicates that *T*_th_ increases nonlinearly with the increase of ratio *K*_43_ of ratios of width and length of N3 and N4 transistors. The transistors N3 and N4 are two equivalent nMOS transistors whose efficient ratios of width and length can be adjusted by some parallel transistors and logic-controlled switches, as shown in Figure 2(b) and 2(c). The logic-control signals are supplied by the threshold temperature setting module. Thus we can program value of *T*_th_ by tuning up the ratios of width and length of N3 and N4, respectively. Because the change of *T*_th_ depends nonlinearly on *K*_43_, additional compensating circuits are required, such as the rightmost 4 branches in Figure 2 (c). Figure 7 shows the dependence of *V*_b_ on temperature with different *K*_43_ parameters. The results indicate that the threshold temperature *T*_th_ of the temperature switch can be programmed by controlling the ratio *K*_43_.

### 2.5. Influence of Mismatch and Vdd variation on T_th_

From Eq. (5), we can see that *K*_43_, M, *V*_B1_ and *V*_B2_ are the main parameters that determine the value *T*_th_, while the influence of size variations of other transistors is not evident. First the *K*_43_ is the ratio of the sizes of N4 and N3 transistors. N4 and N3 are, respectively, composed of several transistors which are integer multiples of the basic unit transistor. When the circuit layout are designed, we can make N3 and N4 transistors match well so that the process variation of *K*_43_ is smaller than 1%. Then M is the ratio of the capacitor C_1_ to the capacitor C_2_. In the modern CMOS process, the process variation of the ratio M can be controlled to be below 1% by the capacitor matching technique. Therefore the process variation of M almost does not influence the value *T*_th_. Finally when a Vdd variation happens, *V*_B1_ and *V*_B2_ change in the same orientation linearly. If Vdd varies within ±10%, the change of (*M* + 1)*V_B_*_2_ − *MV_B_*_1_ can be limited in ±1.5mV with an appropriate value of M. From Eq. (5), the variations of *K*_43_, M and Vdd make *T*_th_ vary within ±1°C.

## 3. Implementation of Temperature Switch

The temperature switch circuit was implemented in a standard 0.35μm CMOS process. The basic temperature switch circuit, the threshold temperature setting module and the sampling and readout module were integrated. The basic temperature switch is analog circuit. The threshold temperature setting module and the sampling and readout module are logic circuits. In order to make the compensation for the *T*_th_ variation effective, the *V*_B1_ and *V*_B2_ bias circuit was placed near the devices in the temperature switch core circuit. Sixteen different *T*_th_s can be set from +45°C to +120°C in a 5°C increment. Simulated results indicates that the setting error of the threshold temperature *T*_th_ is kept within ±2°C in a normal variation range of the process.

## 4. Measurement Result

**Figure 8.**
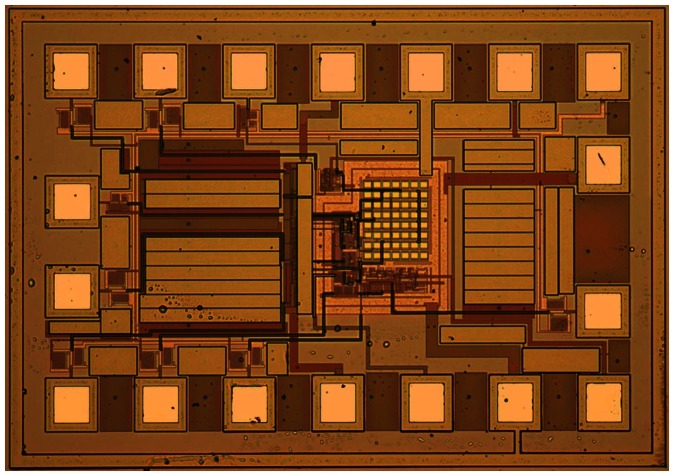
Chip microphotograph of the temperature switch.

**Figure 9.**
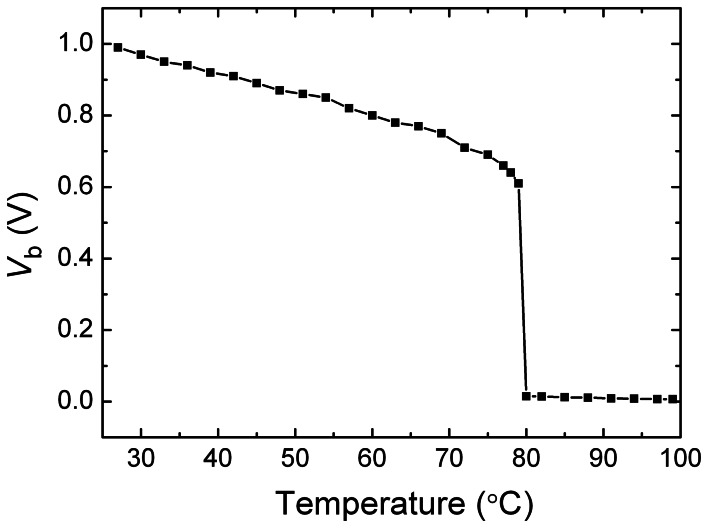
The measured dependence of *V*_b_ on temperature *T*.

**Figure 10.**
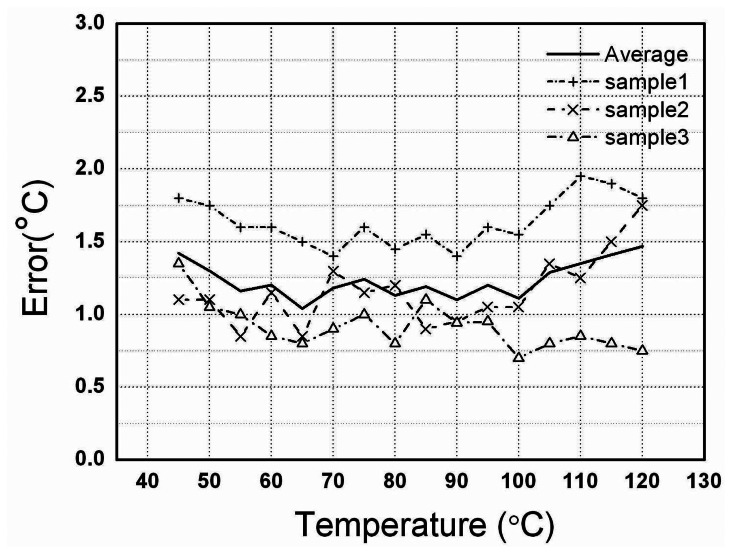
The measured error in the threshold temperatures for 3 samples.

The chip microphotograph is shown in figure 8. The chip core area is only 0.04 mm^2^. After its *T*_th_ was set, we changed the temperature and measured the voltage *V*_b_ at a sampling frequency of 2Hz. Figure 9 gives the measured typical dependence of *V*_b_ on temperature *T*. It shows a very good temperature switch characteristic with the threshold temperature *T*_th_ of 80°C. At the threshold temperature, the sampling and readout module also outputs a switching signal. We programmed the threshold temperature *T*_th_ with external digital codes, and a series of the measured *T*_th_ values could be obtained. Figure 10 shows the measured error in the threshold temperatures for 3 samples. The design parameters and the measured results are presented in table 1. In order to compare the switch with other CMOS temperature switch and temperature sensor reported [1,9], the parameters and the measured results of the CMOS temperature switch and temperature sensor are also listed in table 1. Note that the circuit in [9] is a temperature sensor whereas the presented circuit and the circuit in [1] are temperature switch.

## 5. Conclusion

A novel uncalibrated(need not to be uncalibrated after fabrication) CMOS programmable temperature switch has been presented with extremely low power consumption, high temperature setting accuracy and small chip area. The threshold temperature *T*_th_ could be programmed by changing the efficient ratio of width and length of the MOS transistors. The threshold temperature variation due to process tolerance has been compensated automatically by a floating gate neural MOS circuit and a bias circuit with resetting switches. The circuit has been implemented in a standard 0.35μm CMOS process. Sixteen different *T*_th_s can be set from +45°C to +120°C in 5°C increments. The measurement shows a good consistency in the threshold temperatures for 3 samples. The chip core area is 0.04 mm^2^. Its power consumption is only 3.1μA at 3.3V supply. The temperature switch has the advantages: low power consumption, the programmable threshold temperature, automatic compensation for process tolerance and the controllable hysteresis.

## Figures and Tables

**Table 1.** Measured characteristics of the temperature switch and comparison with the temperature switch and sensor reported.

	This work	[Schinkel]	[Bakker*]
**Supply voltage**	2.3—3.3 V	1.0—1.8V	2.2—5V
**Supply current** T=25°C	3.1μA	13μA	25μA
**Core area**	0.04 mm^2^	0.03 mm^2^	1.5 mm^2^
**Switch temperature**	45—120°C with 5°C increase	128.5°C	–
**Inaccuracy(3**σ**)**	<2°C(typical error)	1.1°C	1°C
**Calibration**	No	No	Yes
**DC supply sensitivity**	1°C/V (2.7V—3.3V)	0.05°C/V	0.1°C/V
**Hysteresis**	0°C	1.2°C	–

* Most of the research of temperature sensor in recent years are focused on high accuracy and they have much higher consumptions. They are not suitable to compared with our design, so we select this earlier paper for a comparison. The circuit was a state-of-the-art, calibrated and chopped, CMOS temperature sensor.
